# A switch in RND3-RHOA signaling is critical for melanoma cell invasion following mutant-BRAF inhibition

**DOI:** 10.1186/1476-4598-10-114

**Published:** 2011-09-14

**Authors:** R Matthew Klein, Paul J Higgins

**Affiliations:** 1 Center for Cell Biology and Cancer Research, MC-165, Albany Medical College, 47 New Scotland Avenue, Albany, NY 12206 USA

## Abstract

**Background:**

The initial use of BRAF targeted therapeutics in clinical trials has demonstrated encouraging responses in melanoma patients, although a rise in drug-resistant cells capable of advancing malignant disease has been described. The current study uses BRAF^V600E ^expressing WM793 melanoma cells to derive data aimed at investigating the molecular determinant of cell invasion following treatment with clinical BRAF inhibitors.

**Findings:**

Small-molecule inhibitors targeting BRAF reduced MEK1/2-ERK1/2 pathway activation and cell survival; yet, viable cell subpopulations persisted. The residual cells exhibited an elongated cell shape, prominent actin stress fibers and retained the ability to invade 3-D dermal-like microenvironments. BRAF inhibitor treatments were associated with reduced expression of RND3, an antagonist of RHOA activation, and elevated RHOA-dependent signaling. Restoration of RND3 expression or RHOA knockdown attenuated the migratory ability of residual cells without affecting overall cell survival. The invasive ability of BRAF inhibitor treated cells embedded in collagen gels was diminished following RND3 re-expression or RHOA depletion. Conversely, melanoma cell movement in the absence of BRAF inhibition was unaffected by RND3 expression or RHOA depletion.

**Conclusion:**

These data reveal a novel switch in the requirement for RND3 and RHOA in coordinating the movement of residual WM793 cells that are initially refractive to BRAF inhibitor therapy. These results have important clinical implications because they suggest that combining BRAF inhibitors with therapies that target the invasion of drug-resistant cells could aid in controlling disease relapse.

## Findings

Cutaneous melanoma is the most lethal skin cancer and its incidence rates continues to rise [1]. Clinical grade small molecule inhibitors targeting BRAF have recently emerged due to its frequent mutational status [2] and vital role in malignancy [3,4]. In particular, a structure-based approach led to the development of PLX-4720, a potent inhibitor of BRAF kinase activity with a V600E mutation [5]. PLX-4720 selectively inhibits MEK1/2-ERK1/2 activation, cell proliferation and xenograph tumor growth using mutant BRAF expressing cell lines [5,6]. PLX-4720 is an analog of the clinically tested PLX-4032 (aka RG7204/Vermurafenib) compound which has demonstrated favorable therapeutic responses [7-9]. Although the durability of PLX-4032 is still under investigation, tumor relapse has been reported [7,8].

A combination of strategies has been suggested to be required for successful therapeutic outcomes in melanoma [10,11]. The addition of an anti-invasive agent to complement targeted BRAF inhibition constitutes an additional therapy that may improve patient outcomes by preventing or delaying the dissemination of drug-resistant clones; however, little is known regarding melanoma invasive strategies following BRAF inhibition. RND3-RHOA cell signaling was identified as a mutant-BRAF regulated pathway [12] that coordinates cell movement [13]. RND3 is an atypical RHO-GTPase [14] that antagonizes RHO-ROCKI signaling [15,16]. Whether this pathway participates in melanoma invasion following BRAF inhibition is unknown.

**Figure 1:**
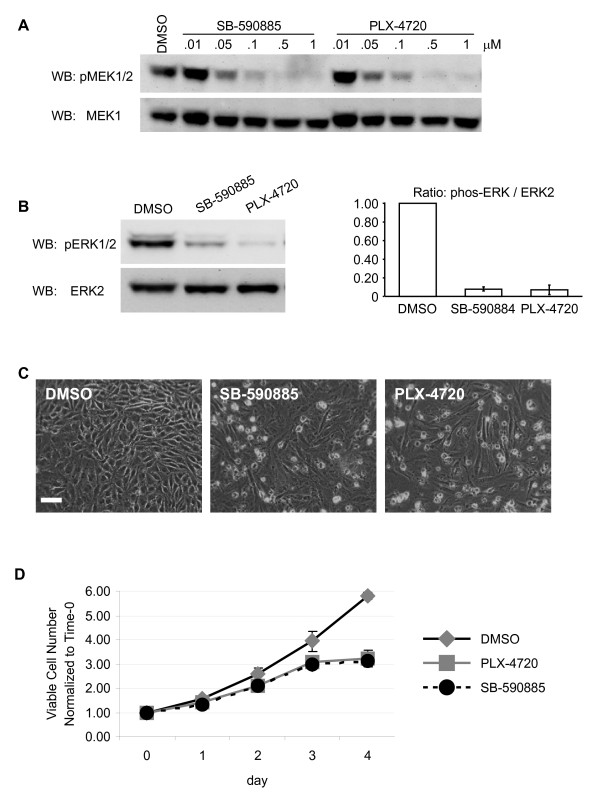
**A sub-population of viable melanoma cells persist following BRAF inhibition**. Invasive WM793 human melanoma cell layers treated 48 h with DMSO or pharmacological inhibitors targeting total BRAF (SB-590885) or mutant BRAF (PLX-4720) from B-Bridge Int. (Cupertino, CA). **A**) Cell layers were treated with increasing concentration (0.01, 0.05, 0.1, 0.5, 1.0 μM) of inhibitors, cell lysates were generated and analyzed by Western blot using antibodies from Cell Signaling Technology (Danvers, MA); phos-MEK1/2 (9121) and total MEK1 (9124). **B**) Western blot analysis of lysates from cells treated with 0.5 μM SB-590885, 0.5 μM PLX-4720 or DMSO, phos-ERK1/2 (sc7383) and total ERK2 (sc154) antibodies from Santa Cruz Biotech (Santa Cruz, CA). Graphed is the mean ± SD of phos-ERK1/2:ERK2 ratio from 3 experiments with the DMSO condition set to one. **C**) Micrographs depicting cell layers treated with inhibitors, as described above. **D**) Time-course indicating viable melanoma cells following BRAF inhibitor treatments, as determined by toludine blue staining; Graph shows average ± SD).

**Figure 2:**
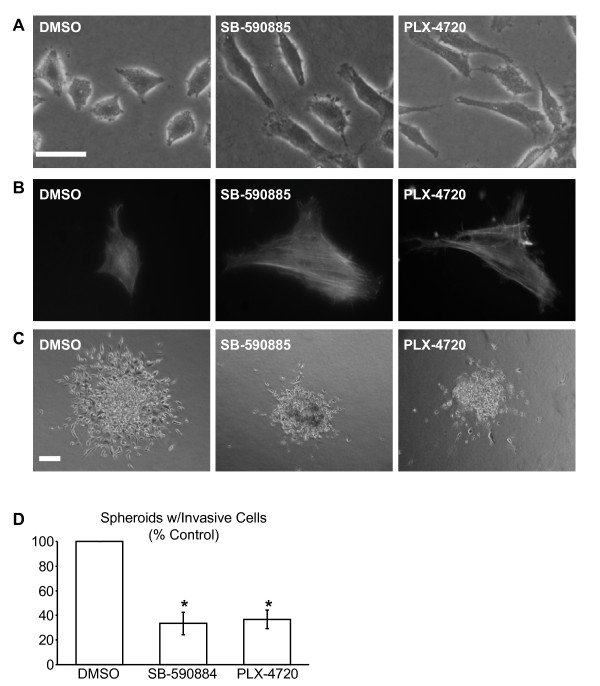
**Phenotypic characterization of cells treated with pharmaceutical BRAF inhibitors**. **A, B**) Melanoma cells treated ± inhibitors 48 h with 0.5 μM SB-590885, 0.5 μM PLX-4720 or DMSO. Adherent cells were trypsinized and plated on top a collagen gel [13] for an additional 24 h in the continued presence of inhibitors. **A**) Cell morphology of control (DMSO) or BRAF inhibitor treated cultures, visualized using phase-contrast microscopy. **B**) Fluorescent visualization of F-actin organization, TRITC-conjugated phalloidin (P1951) was from Sigma-Aldrich (St. Louis, MO). **C**) Phase contrast images of melanoma spheroids that were embedded in a collagen gel for three days ± inhibitors [10]. **D**) Quantitation of spheroids, shown in (C), that contains cells insensitive to BRAF inhibitors. (Graph shows average ± SD; *P < 0.05).

Human WM793 melanoma cells express BRAF^V600E ^[17] and are hemizygously deleted for PTEN with a mutation (W274X) in the remaining allele [18]. Targeted knockdown of BRAF rather than ARAF or CRAF reduces MEK1/2-ERK1/2 phosphorylation (Additional file 1, Figure S1). Likewise, pharmaceutical inhibition of BRAF elicited dose-dependent reductions in MEK1/2 phosphorylation (Figure 1A). ERK1/2 phosphorylation decreased ~92% in cells treated with either 0.5 μM SB-590885, a potent inhibitor of total BRAF [19] or 0.5 μM PLX-4720, the BRAF^V600E ^selective inhibitor (Figure 1B). Interestingly, numerous cells remained attached and well spread following inhibitor treatments (Figure 1C), suggesting survival may not have been negatively impacted. Viable cells were identified following 96 h incubations with either SB-590885 or PLX-4720 (Figure 1D). Cell viability was further evaluated after re-plating onto non-fibrillar collagen gels, in the continued presence of the drugs. BRAF inhibition led to dramatic morphological changes; cells appeared elongated and less refractive compared to control cells (Figure 2A). Viable cells were identified in ~59% of SB-590885 and ~63% of PLX-4720 treated cultures (Additional file 2, Figure S2). These data indicate that melanoma cells harboring a BRAF^V600E ^mutation can survive despite reductions in BRAF activation of the MEK-ERK signaling cascade.

BRAF knockdown alters cytoskeletal architecture and cell shape [12]; therefore, it was important to assess whether alterations in F-actin also accompanied pharmaceutical BRAF inhibition. Control cells plated on collagen gels exhibited diffuse microfilament staining patterns with thin cortical fibers (Figure 2B). In contrast, prominent F-actin stress fibers typified BRAF inhibitor treated cells (Figure 2B); these stress fiber traversed the cell body often terminating in large bundles at the cell membrane. Cell elongation and prominent actin stress fibers, therefore, correlate with viable melanoma cells in the presence of BRAF inhibitors.

To determine if drug insensitivity occurred in a more physiological setting melanoma spheroids embedded into a 3-D collagen gel, to recapitulate a stromal-like environment [10], were treated with inhibitors in complete medium. Controls cultures invaded the surrounding extracellular matrix (Figure 2C). SB-590885 and PLX-4720 treatment attenuated invasive outgrowth (Figure 2C), although some spheroids were surrounded with elongated cells that invaded the surrounding microenvironment (Figure 2C). Invasive cells were evident in 33% and 36% of spheroid structures treated with SB-590885 and PLX-4720, respectively, (Figure 2D) clearly signifying that some cells can invade a 3-D microenvironment following pharmaceutical BRAF inhibition.

**Figure 3:**
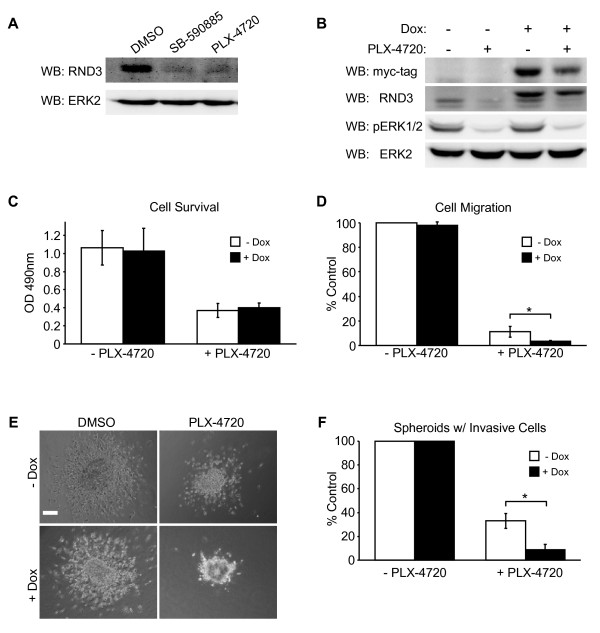
**RND3 downregulation participates in the invasion of melanoma cells insensitive to BRAF inhibition**. **A**) Immunoblot of lysates from cells treated 48 h with 0.5 μM SB-590885, 0.5 μM PLX-4720 or DMSO using antibodies directed toward RND3 (05-723 from Upstate Biotech Inc.) and total ERK2. **B-F**) Inducible expression of myc-tagged wild-type RND3 in WM793 melanoma cells cultured in complete medium ± doxycycline (Dox) in the absence or presence of PLX-4720. **B**) Western blot of cell lysates using antibodies specific for myc-tag, RND3, phos-ERK1/2 and ERK2. **C**) The viability of cells, treated as in (A), cultured four days was monitored by Toludine blue staining, graphed are the results from one experiment performed in triplicate. **D**) Cells, treated as in (A), plated in serum-free medium into the upper well of a Boyden migration chamber pre-coated with fibronectin + collagen mixture (10 μg/ml). The lower well contained complete medium, to stimulate cell migration. Sixteen hours later, cells that migrated to the insert bottom were labeled with Hoescht Dye and counted by fluorescent microscopy. **E**) Micrographs depicting invasive outgrowth of spheroids, treated as indicated, embedded inside a 3-D collagen gel. **F**) Quantitation of the number of spheroids that contained invasive cells, as depicted in (E). All experiments were performed in triplicate. The graphs presented represent mean ± SD, statistical significance determined using Student's *t*-test and *P*-values < 0.05 considered significant (*).

Alterations in BRAF regulated signaling pathways that could affect actin organization and melanoma invasion were then evaluated. RND3 expression is increased in invasive melanoma cells expressing BRAF^V600E ^[13] and is a known regulator of actin organization [14]. Therefore, we assessed whether BRAF inhibitors had an effect on RND3. Western blot analysis indicated that reduced RND3 expression accompanied pharmaceutical inhibition of BRAF (Figure 3A). We then constructed a doxycycline-inducible myc-tagged RND3 expression system to determine if reduced RND3 expression was required for melanoma invasion in the presence of BRAF inhibitors. This system allowed for sustained RND3 expression despite reduced BRAF signaling to ERK1/2 (Figure 3B). Inducible expression of RND3 did not affect ERK1/2 activation or inhibition (Figure 3B). The functionality of ectopic RND3 expression was confirmed by microscopic evaluation of F-actin staining. RND3 over-expression attenuated the formation of actin stress fibers in response to BRAF inhibition (Additional file 3, Figure S3A), although, sustained RND3 expression did not prevent increases in cofilin phosphorylation which accompanied BRAF inhibition (Additional file 3, Figure S3B). The effect increased RND3 expression had on cell growth was then assessed. Induced RND3 expression did not affect basal growth nor did it alter reductions in cell growth associated with BRAF inhibition (Figure 3C). It was important, therefore, to evaluate the effect restoring RND3 expression had on the migration of BRAF inhibitor treated cells. BRAF inhibition reduced cell migration by approximately 85% (Figure 3D). Ectopic RND3 did not affect basal cell migration, although, its sustained expression significantly diminished the migration of PLX-4720 treated cells (Figure 3D). To determine if reduced RND3 expression was required for the invasion of BRAF inhibitor treated cells, we monitored the invasive outgrowth of PLX-4720 treated spheroids embedded into collagen gels in the presence or absence of RND3. Sustained RND3 expression significantly reduced (~24%) the frequency of invasive cells evident in PLX-4720 treated cultures (Figure 3C and 3D), whereas, it did not affect non-treated spheroids (Figure 3C and 3D). Thus, reduced RND3 expression supports melanoma invasion following BRAF inhibition.

**Figure 4:**
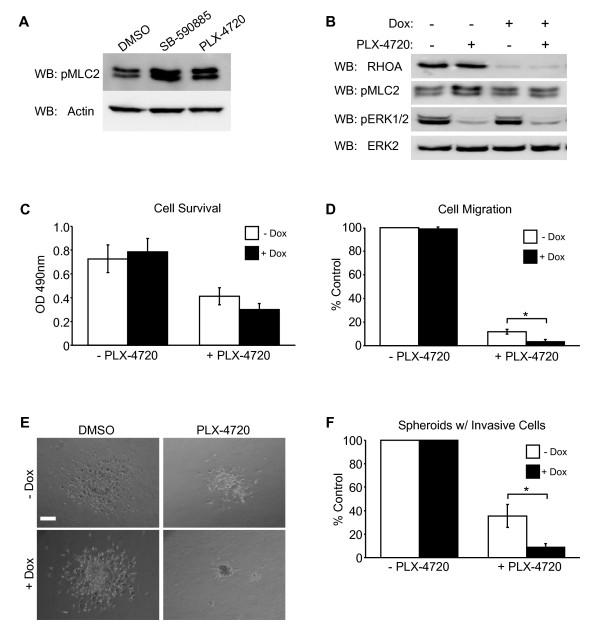
**Endogenous RHOA depletion antagonizes the invasion of melanoma cells that are insensitive to BRAF inhibition**. **A**) Lysates generated from cells treated 48 h with 0.5 μM SB-590885, 0.5 μM PLX-4720 or DMSO, subjected to Western blot analysis using antibodies specific for phosphorylated (Thr18/Ser19) myosin light chain 2 (Cell Signaling #3674) and actin. **B**) Dox-inducible RHOA shRNA (target sequence: ATGGAAAGCAGGTAGAGTT) melanoma cells were treated ± 0.5 μM PLX-4720 for 48 h. Cell lysate analyzed by Western blot for RHOA (sc418), phos-MLC2, phos-ERK1/2 and ERK2. **C**) The viability of cells, treated as in (**B**), monitored by toludine blue staining, representative graph of the results from one experiment performed in triplicate. **D**) Boyden chamber analysis of cell migration (as described in Figure 3D) following treatments shown in (**B**). Graph indicates average number of migrated cells ± SD. **E**) Micrographs of invasive outgrowth associated with inducible RHOA shRNA spheroids in the presence or absence of mutant BRAF inhibition. **F**) Quantitation (average ± SD) of the number of spheroids, as depicted in (**E**), that harbor drug refractive cells. All experiments were performed in triplicate. Statistical significance determined using Student's *t*-test and *P*-values < 0.05 were considered significant and represented by *.

In invasive melanoma, RND3 expression regulates actin organization through RHOA [12]. To investigate whether BRAF inhibitors enhanced RHOA-dependent signaling, we monitored the activation of the downstream RHOA-ROCK1/2 effector, myosin regulatory light chain. Treatment of cells with PLX-4720 or SB-590885 resulted in increased phosphorylation of myosin light chain 2 (Figure 4A), suggestive of enhanced RHOA signaling. To establish whether RHOA was required for melanoma invasion despite BRAF inhibition, RHOA knockdown cells were generated. Inducible depletion of RHOA by shRNA in the absence or presence of PLX-4720 was confirmed by Western blot (Figure 4B). RHOA knockdown did not affect drug inhibition of ERK phosphorylation, although, depletion of RHOA was functional as observed by the prevention of increased myosin light chain 2 phosphorylation (Figure 4B) and actin stress fiber formation following BRAF inhibition (Additional file 4, Figure S4A). Knockdown of RHOA did not impact the increase in cofilin phosphorylation (Additional file 4, Figure S4B) or reduction in cell growth that accompanied BRAF inhibition (Figure 4C). RHOA depletion (Figure 4D), and ROCKI/II inhibition (Additional file 5, Figure S5), attenuated cell migration in PLX-4720 treated cultures. The requirement for RHOA in the 3-D invasive outgrowth of melanoma spheroids in the presence of PLX-4720 was then evaluated. Depletion of RHOA alone did not affect invasive outgrowth (Figure 4E and 4F). However, the combination of PLX-4720 treatment and RHOA knockdown further reduced the number of spheroids that contained invasive cells by ~26% (Figure 4E and 4F). These results demonstrate that RHOA participates in residual melanoma cell invasion following pharmaceutical BRAF inhibition.

Cancer cell resistance to cytotoxic agents is a common and severe therapeutic impediment that can lead to the reemergence of malignant tumors. This study demonstrates that a subpopulation of melanoma cells can survive and invade a dermal-like extracellular matrix, despite BRAF inhibitor treatments. These findings agree with others who have shown that melanoma cell lines expressing a BRAF^V600E ^mutation can established resistance to BRAF inhibitors in culture [20,21] as well as a xenograph mouse model [22]. Moreover, despite encouraging clinical trial outcomes using PLX-4032 [7,8], the development of BRAF inhibitor resistant cells has been reported [23-25]. Collectively these studies advocate for the preparation of therapies that prevent the development of drug-insensitive clones or block the ability of these cells to spread and metastasize.

The present work identifies factors that facilitate the residual invasion of BRAF^V600E ^expressing melanoma cells after pharmaceutical BRAF inhibition by employing 2-D and more physiological 3-D preclinical models. Initially, an elongated cell shape with prominent actin stress fibers were identified as phenotypic markers of viable cells following BRAF inhibition. Importantly, the correlation between cytoskeletal remodeling and drug insensitivity does not implicate prominent actin stress fibers as a predictive factor or "biomarker" for melanoma resistance to BRAF inhibition. The development of actin stress fibers more closely reflects enhanced RHOA pathway signaling. The current study identifies novel roles for RND3 and RHOA in the movement but not growth or survival of melanoma cells treated with BRAF inhibitors. These findings suggest that BRAF inhibition invokes a switch in the utilization of the RND3-RHOA signaling pathway. Accordingly, RND3 expression and suppressed RHOA signaling appear to be important for normal melanoma cell movement, whereas RND3 downregulation and enhanced RHOA signaling are critical in BRAF-inhibitor treated cells. Collectively, these data demonstrate that interfering with signaling pathways which facilitate the invasion of drug-resistant tumor cells may represents a cytostatic therapy that could complement BRAF inhibitor therapeutics.

## List of abbreviations

None

## Competing interests

The authors declare that they have no competing interests.

## Authors' contributions

RMK: principal investigator of this project, contributed to overall project conception, design, data acquisition, data analysis and manuscript preparation. PJH: contributed to the project design and edited the manuscript. All authors read and approved the final manuscript.

## Author's information

RMK is Research Associate in the Center for Cell Biology and Cancer Research at Albany Medical College. PJH is Professor and Co-Director of the Center for Cell Biology and Cancer Research at Albany Medical College.

## Supplementary Material

Additional file 1 **BRAF supports elevated ERK1/2 phosphorylation in WM793 melanoma cells**. WM793 melanoma cells treated 72 h with siRNA from Dharmacon targeting ARAF (L-003563-00), BRAF (L-003460-00), CRAF (L-006301-00) or non-targeting control (D-001210-01) using oligofectamine. Cell lysates were generated and immunoblotted using antibodies from Santa Cruz Biotech (Santa Cruz, CA): ARAF (sc407), BRAF (sc5284), CRAF (sc133), phos-ERK1/2 (sc7383) and total ERK2 (sc154). Click here for file

Additional file 2 **Viable melanoma cells persist following BRAF inhibition**. Invasive WM793 human melanoma cells treated with pharmacological inhibitors targeting total BRAF (0.5 μM SB-590885), mutant BRAF (0.5 μM PLX-4720) or equal volume DMSO. Cell layers incubated ± inhibitors 48 h then seeded on top a collagen gel an additional 24 h in the continued presence of inhibitors. **A**) Representative images from dual-fluorescent cell viability assay; Calcein-AM - live cells, EthD-1 - dead cells (Invitrogen). **B**) Quantitation of live/dead cells counted on collagen gels, as shown in (**A**). Graph shows mean ± SD % of live cells counted from three independent experiments (n = 300). Click here for file

Additional file 3 **RND3 restoration disrupts PLX-4720 induced actin stress fiber formation**. **A**) Micrographs depicting F-actin organization in Dox-inducible RND3 expressing WM793 melanoma cells treated with 0.5 μM PLX-4720 or equal volume DMSO. Cells incubated ± inhibitors 48 h were then seeded on top a collagen gel an additional 24 h in the continued presence of inhibitors. Cell layers were then were fixed and processed to visualize F-actin organization. **B) **Cell lysates generated and immunoblotted using antibodies from Cell Signaling Tech. (Danvers, MA): phospho-Cofilin (3311) and Santa Cruz Biotech (Santa Cruz, CA): total ERK2 (sc154). Click here for file

Additional file 4 **RHOA is required for PLX-4720 induced actin stress fiber formation**. **A**) Micrographs depicting F-actin organization in Dox-inducible RHOA shRNA expressing WM793 melanoma cells treated with 0.5 μM PLX-4720 or equal volume DMSO. Cells incubated ± inhibitors 48 h were then seeded on top a collagen gel an additional 24 h in the continued presence of inhibitors. Cell layers were then were fixed and processed to visualize F-actin organization. **B) **Cell lysates generated and immunoblotted using antibodies from Cell Signaling Tech. (Danvers, MA): phospho-Cofilin (3311) and Santa Cruz Biotech (Santa Cruz, CA): total ERK2 (sc154). Click here for file

Additional file 5 **ROCKI/II are utilized for residual cell migration following PLX-4720 treatment**. Cells treated 48 hours ± 0.5 μM PLX-4720 were plated into the upper well of a Boyden migration chamber pre-coated with a fibronectin + collagen mixture (10 μg/ml) in the absence or presence of 5 μM Y27632, a ROCKI/II inhibitor. The lower well contained complete medium in the absence or presence of inhibitors, as indicated, to stimulate cell migration. Sixteen hours later, cells that migrated to the insert bottom were labeled with Hoescht Dye and counted by fluorescent microscopy. **A**) Micrographs depicting migrated cells. **B**) Graph indicates average number of migrated cells ± SD. Statistical significance (*) determined by Student's *t*-test (*P*-value = .048). Click here for file
