# Supplementary figures and images for: A switch in RND3-RHOA signaling is critical for melanoma cell invasion following mutant-BRAF inhibition
Source: Mol Cancer. 2011 Sep 14;10:114. doi: 10.1186/1476-4598-10-114 (PMC3180434; doi:10.1186/1476-4598-10-114)

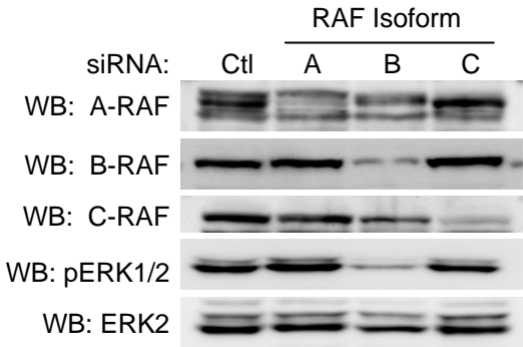

Supplement: Additional file 1 — BRAF supports elevated ERK1/2 phosphorylation in WM793 melanoma cells. WM793 melanoma cells treated 72 h with siRNA from Dharmacon targeting ARAF (L-003563-00), BRAF (L-003460-00), CRAF (L-006301-00) or non-targeting control (D-001210-01) using oligofectamine. Cell lysates were generated and immunoblotted using antibodies from Santa Cruz Biotech (Santa Cruz, CA): ARAF (sc407), BRAF (sc5284), CRAF (sc133), phos-ERK1/2 (sc7383) and total ERK2 (sc154). [file 1476-4598-10-114-S1.PDF]

**A**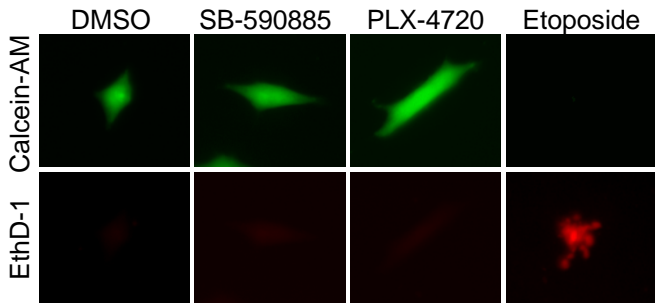**B**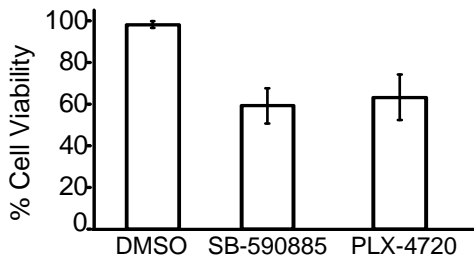

Supplement: Additional file 2 — Viable melanoma cells persist following BRAF inhibition. Invasive WM793 human melanoma cells treated with pharmacological inhibitors targeting total BRAF (0.5 μM SB-590885), mutant BRAF (0.5 μM PLX-4720) or equal volume DMSO. Cell layers incubated ± inhibitors 48 h then seeded on top a collagen gel an additional 24 h in the continued presence of inhibitors. A) Representative images from dual-fluorescent cell viability assay; Calcein-AM - live cells, EthD-1 - dead cells (Invitrogen). B) Quantitation of live/dead cells counted on collagen gels, as shown in (A). Graph shows mean ± SD % of live cells counted from three independent experiments (n = 300). [file 1476-4598-10-114-S2.PDF]

**A**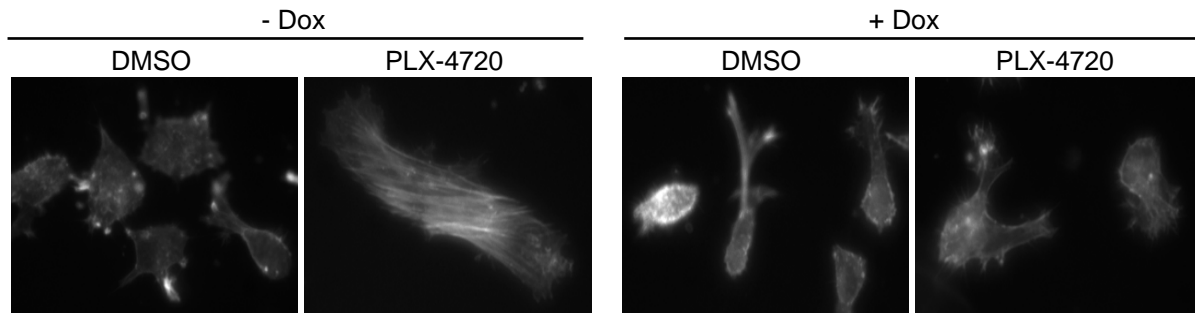**B**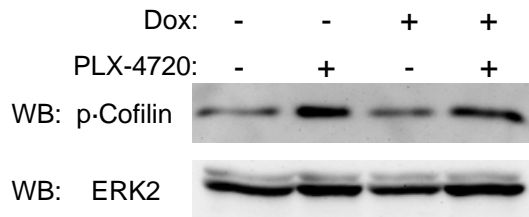

Supplement: Additional file 3 — RND3 restoration disrupts PLX-4720 induced actin stress fiber formation. A) Micrographs depicting F-actin organization in Dox-inducible RND3 expressing WM793 melanoma cells treated with 0.5 μM PLX-4720 or equal volume DMSO. Cells incubated ± inhibitors 48 h were then seeded on top a collagen gel an additional 24 h in the continued presence of inhibitors. Cell layers were then were fixed and processed to visualize F-actin organization. B) Cell lysates generated and immunoblotted using antibodies from Cell Signaling Tech. (Danvers, MA): phospho-Cofilin (3311) and Santa Cruz Biotech (Santa Cruz, CA): total ERK2 (sc154). [file 1476-4598-10-114-S3.PDF]

**A**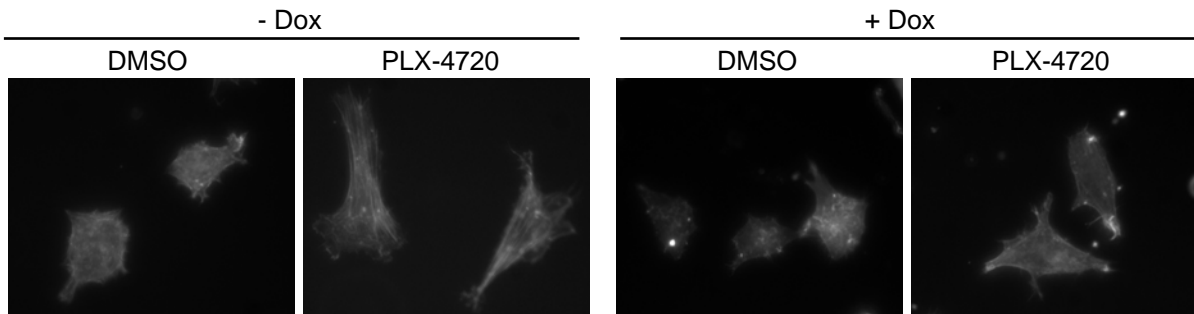**B**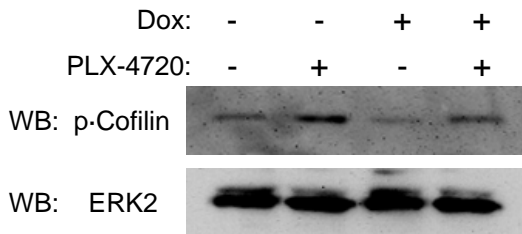

Supplement: Additional file 4 — RHOA is required for PLX-4720 induced actin stress fiber formation. A) Micrographs depicting F-actin organization in Dox-inducible RHOA shRNA expressing WM793 melanoma cells treated with 0.5 μM PLX-4720 or equal volume DMSO. Cells incubated ± inhibitors 48 h were then seeded on top a collagen gel an additional 24 h in the continued presence of inhibitors. Cell layers were then were fixed and processed to visualize F-actin organization. B) Cell lysates generated and immunoblotted using antibodies from Cell Signaling Tech. (Danvers, MA): phospho-Cofilin (3311) and Santa Cruz Biotech (Santa Cruz, CA): total ERK2 (sc154). [file 1476-4598-10-114-S4.PDF]

**A**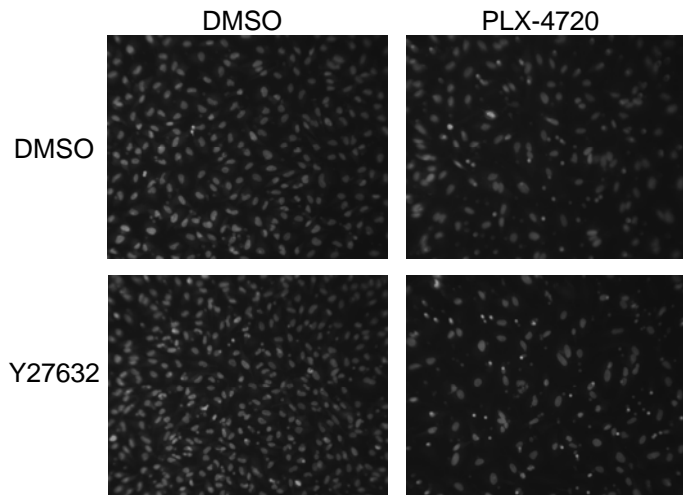**B**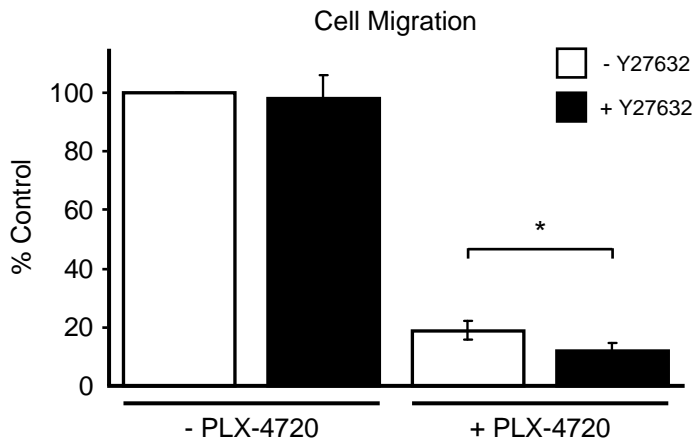

Supplement: Additional file 5 — ROCKI/II are utilized for residual cell migration following PLX-4720 treatment. Cells treated 48 hours ± 0.5 μM PLX-4720 were plated into the upper well of a Boyden migration chamber pre-coated with a fibronectin + collagen mixture (10 μg/ml) in the absence or presence of 5 μM Y27632, a ROCKI/II inhibitor. The lower well contained complete medium in the absence or presence of inhibitors, as indicated, to stimulate cell migration. Sixteen hours later, cells that migrated to the insert bottom were labeled with Hoescht Dye and counted by fluorescent microscopy. A) Micrographs depicting migrated cells. B) Graph indicates average number of migrated cells ± SD. Statistical significance (*) determined by Student's t-test (P-value = .048). [file 1476-4598-10-114-S5.PDF]
